# Investigation of the Correlation between Postherpetic Itch and Neuropathic Pain over Time

**DOI:** 10.1155/2018/9305126

**Published:** 2018-12-02

**Authors:** Rie Ishikawa, Masako Iseki, Rie Koga, Eiichi Inada

**Affiliations:** ^1^Department of Anesthesiology and Pain Medicine, Juntendo University Faculty of Medicine, 1-3 Hongo 3-chome, Bunkyo-ku 113-8431, Japan; ^2^Department of Anesthesia and Pain Medicine, Hachinohe Heiwa Hospital, 4-6 Minatotakadai 2-chome, Hachinohe 031-8545, Japan; ^3^Department of Pain Medicine, Sata Hospital, 4-28 Watanabedori 2-chome, Chuo-ku 810-0004, Japan

## Abstract

Postherpetic itch (PHI), or herpes zoster itch, is an intractable and poorly understood disease. We targeted 94 herpes zoster patients to investigate their pain and itch intensities at three separate stages of the condition (acute, subacute, and chronic). We used painDETECT questionnaire (PDQ) scores to investigate the correlation between PHI and neuropathic pain. Seventy-six patients were able to complete follow-up surveys. The prevalence of PHI was 47/76 (62%), 28/76 (37%), and 34/76 (45%) at the acute, subacute, and chronic stages, respectively. PHI manifestation times and patterns varied. We investigated the relationship of PHI with neuropathic pain using the visual analog scale (VAS), which is a measure of pain intensity, and the PDQ, which is a questionnaire used to evaluate the elements of neuropathic pain. The VAS and PDQ scores did not differ significantly between PHI-positive and PHI-negative patients. A large neuropathic component was not found for herpes zoster itch, suggesting that neuropathic pain treatments may not able to adequately control the itch. Accordingly, we suggest that a more PHI-focused therapy is required to address this condition.

## 1. Introduction

We have been investigating postherpetic pains focusing the nature of such pains [1], during which we have noticed many patients suffering form postherpetic pains also suffer from itch. Our clinical experiences with both the itch and pain indicate that the treatments for postherpetic pain have little effect on itch, which sometimes persists being refractory to any treatments. This itching is termed postherpetic itch (PHI) and is clinically experienced in the following four ways: (1) as a transient itch at the time of rash healing, (2) as itching concomitant with pain, (3) as a sensation of itching that intensifies as pain subsides, and (4) as the sole persistent symptom. PHI has been classified as a neuropathic itch resulting from inflammation and nerve damage in a similar manner to pain; however, much remains unclear about its etiology. The pathology of neuropathic itch at the cellular/molecular level is yet to be elucidated [2]. In an epidemiological study in herpes zoster patients conducted by Oaklander et al., itching occurred in 9% of patients at the acute stage (before or during their zoster infection), and the prevalence of PHI ranged from 30% to 58% at the chronic stage in patients who had developed PHN [3]. However, chronological changes in itching have not been investigated in detail.

PHI is an intractable condition for which no therapeutic approach has been established. Reports on PHI are limited to clinical experiences, and few case studies exist. PHI has not been resolved in many cases where therapy for neuropathic pain has been applied; such resolutions should have been achieved if PHI and neuropathic pain shared a similar pathology. PHI and neuropathic pain are considered to have different pathologies. Accordingly, we set out to investigate any neuropathic components using the painDETECT questionnaire (PDQ) [4, 5] and to evaluate its relationship with PHI. We also investigated the correlation between pain and itch intensity.

## 2. Materials and Methods

This study involved a survey of 94 patients receiving treatment for herpes zoster-related pain at our pain clinic on an outpatient basis between June 2013 and January 2015. They were studied beginning between 8 days and 1 month after the onset of herpes zoster. In this prospective study, the patients were surveyed using the PDQ and a visual analog scale (VAS) to evaluate pain intensity and an 11-point scale (0 to 10) to determine itch intensity. We used different scales to assess pain and itch. Pain was assessed by a VAS (100 mm), and itch was assessed by an 11-point numeric scale because, in our hospital, pain assessment is routinely performed using a 100 mm VAS. In addition, the 11-point itching scale was used for simplicity.

**Figure 1:**
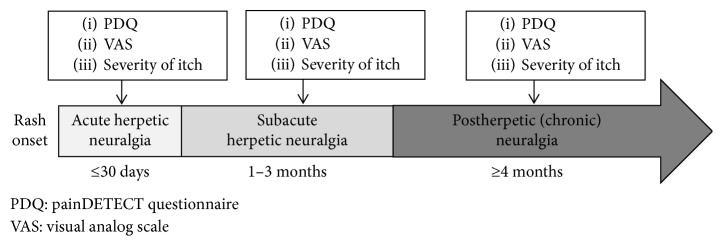
Protocol.

Patients were asked to complete the PDQ at the start of treatment, and the VAS score and itch intensity were determined at the same time. The patients completed follow-up surveys over a subsequent 6-month period. Patients who did not attend the clinic for follow-up visits because their condition had resolved were surveyed by a telephone or through a postal questionnaire. For each follow-up survey, patients were categorized as being at one of these three stages: acute, subacute, or chronic. Their PDQ, itch intensity, and VAS scores were determined for each stage (Figure 1). The stages were determined in accordance with the following definitions established by Dworkin et al.: the acute stage was regarded as the period up to 30 days after the zoster onset, the subacute stage was regarded as the period between 31 and 90 days (1 and 3 months) after the zoster onset, and the chronic stage was regarded as the period beginning 4 months after the zoster onset [6]. Patients are likely to be influenced by the state of the rash in the first week; accordingly, the first week after the zoster onset was omitted from the period regarded as the acute stage (up to 30 days after the onset).

A score of 1 point or greater on the itch intensity assessment scale was taken to indicate PHI, and PHI prevalence and manifestations were investigated for each stage of the illness.

For each stage and PHI status, the results for age, sex, zoster site, total VAS score, and total PDQ score were evaluated. Itch severity was assessed as described by Oaklander et al., with a score on the itch-intensity assessment scale between 1 and 3 points representing a grade of “mild,” a score between 4 and 7 points representing a grade of “moderate,” and a score of 8 points or greater representing a grade of “severe” [3].

We also investigated correlations between itch intensity and VAS and PDQ scores. Furthermore, chronic stage patients with a VAS score above 30 mm were regarded as having PHN, and we investigated the association between PHI status and PHN status.

Statistical analyses were performed using SPSS Statistics 22.0 for Windows (IBM Corp., Japan).

## 3. Results

Seventy-six of the 96 target patients were able to complete the follow-up surveys for 6 months. The prevalence of PHI tended to be high in patients with craniocervical herpes zoster and did not differ with age (Table 1). Therapy regimen details are presented in Table 2.

The prevalence of PHI (by stage) was 47/76 (62%) at the acute stage, 28/76 (37%) at the subacute stage, and 34/76 (45%) at the chronic stage (Table 3). The median itch intensity was 4 points at the acute stage, 2 points at the subacute stage, and 2 points at the chronic stage. The breakdown for itch severity in patients (%) was as follows: the “mild” grade was the most common, with 49% at the acute stage, 86% at the subacute stage, and 79% at the chronic stage, while the “severe” grade (a score of 8 or more on the assessment scale) was observed in 23% at the acute stage, 4% at the subacute stage, and 6% at the chronic stage (Table 3).

PHI patients showed varied itch manifestation times and patterns. Itching manifested across all stages beginning soon after the zoster onset (Table 4). The proportion of patients experiencing itching in all stages was 16%. The proportion of patients experiencing itching in the acute stage only was 22%. The proportion of patients experiencing itching in both the acute and subacute stages was 12%. The proportion of patients experiencing itching in both the acute and chronic stages was 12%. The number of patients with persistent itching after the resolution of pain was 0 (0%) in the acute stage, 3 (11%) in the subacute stage, and 6 (18%) in the chronic stage.

**Figure 2:**
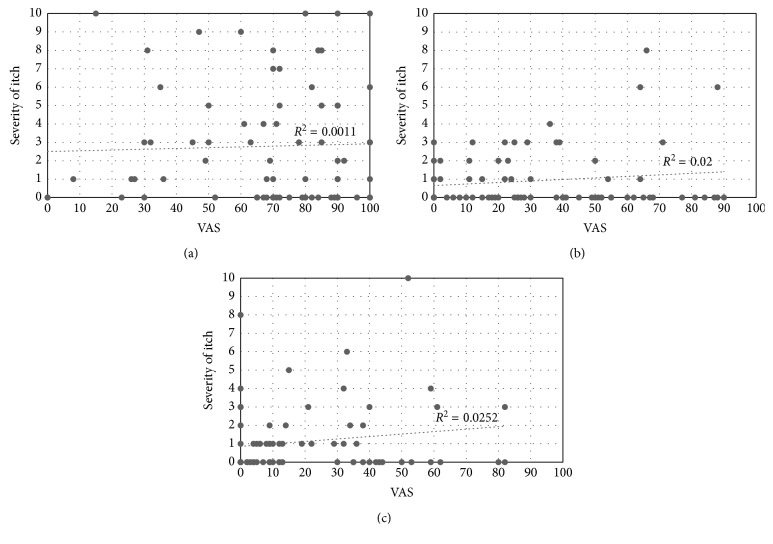
Correlation between itch severity and VAS: (a) acute stage; (b) subacute stage; (c) chronic stage.

**Figure 3:**
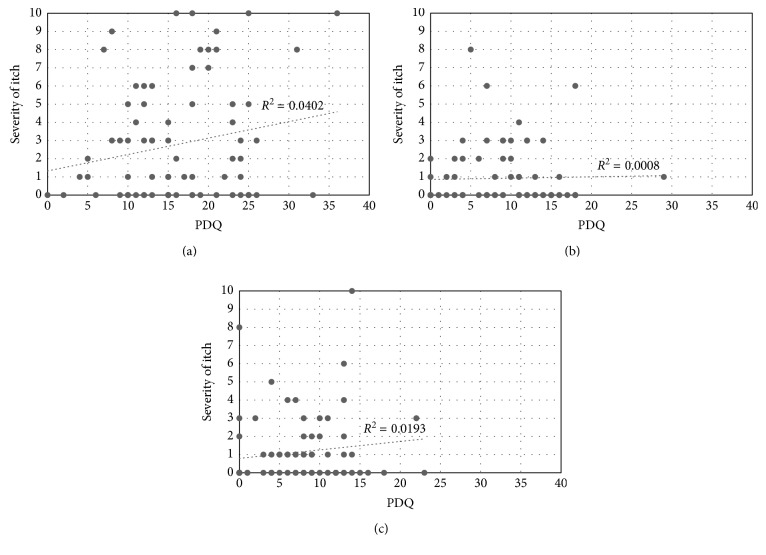
Correlation between itch severity and PDQ: (a) acute stage; (b) subacute stage; (c) chronic stage.

PHN was observed in 25/76 patients (33%); 11 of these patients were PHI positive (this subset accounted for 44% of PHN patients) (Table 5). VAS and PDQ scores are presented by the stage and PHI status in Table 6. VAS and PDQ scores did not differ between PHI-positive and PHI-negative patients at any stage (Table 6). VAS scores and itch intensities showed no correlation (Figure 2). PDQ scores and itch intensities showed a moderate positive correlation in the acute stage but no correlation at other stages (Figure 3).

## 4. Discussion

In their study, Oaklander et al. [3] reported itch prevalence of 17% and 30–58% in herpes zoster patients at a pain management center in the acute (before or during the zoster onset) and chronic (3 months after the zoster onset) stages, respectively. However, in our study, the corresponding prevalence results were 62% in the acute stage, 37% in the subacute stage, and 45% in the chronic stage. We found that itching occurred more widely across the entire observation period. The difference compared with the findings of Oaklander et al. was particularly pronounced for patients with itching in the acute stage (up to 30 days after the zoster onset). We also investigated itch manifestation times and found that itching manifested across all stages from soon after the zoster onset and with various patterns. The most common timing for itch manifestation was in the acute stage only (22%); the proportion of patients experiencing itching in all stages was 16%. Evidently, the itching was transient and resolved as the rash disappeared, but the itch also persisted in many cases. Itch manifestation times varied, with some patients experiencing itching only in the subacute stage or only in the chronic stage. Herpes zoster patients often do not complain of itching during consultations even though they are aware of it as a subjective symptom; accordingly, itching is an issue that needs to be addressed by the consulting physician.

Itch intensity was assessed using an 11-point scale. Combined findings (for severity) of mild to moderate itching were greatest in the acute stage; patients with mild itching still accounted for the greatest proportion even after including patients with severe itching. The patients had presented at our clinic with concomitant pain from the acute stage. Accordingly, treatment for pain was started at an early stage after the zoster onset, with some patients receiving medication for neuropathic pain and nerve blockers. Patients also received medication for neuropathic pain in the subacute and chronic phases. We consider that this medication may have been effective, to a certain extent, against itching not associated with pain. Previous studies have shown that PHI responds well to treatment with an antihistamine agent and concomitant antiepileptic medication [7]; it has also been demonstrated that epidural infusions of bupivacaine and clonidine [8] and stellate ganglion blocks [9] have efficacy against PHI. However, severe itching has persisted in some cases regardless of treatment, with PHI proving intractable against the medications.

Oaklander et al. investigated patients experiencing PHN at least 1 month after zoster disappearance and found that 12.8% of patients still experienced severe itching [3]. Conversely, in our study, the proportion of patients with severe itching was high in the acute stage, at 23%, and low in the subacute and chronic stages, at 4% and 6%, respectively. We treated patients for zoster-associated pain from an early stage after the zoster onset, and we consider that this treatment may have contributed to alleviation of the itching.

PHI is reported to be more likely to occur in cases where herpes zoster affects the head, face, and neck rather than the truck and limbs, and its incidence is reportedly independent of age and sex [3]. Similarly, in our study, PHI occurred relatively frequently for patients with craniocervical herpes zoster, and PHI status showed no age bias. Sex differences were difficult to evaluate in our study because the study population contained a disproportionately large number of women; further investigations with a greater number of patients are needed. Physicians treating patients with craniocervical herpes zoster should pay attention to itching.

We now turn to the relationship between pain and itching in herpes zoster. Oaklander et al. found a slight association between pain and itching [3]. However, we found no correlation between VAS scores and itch intensities. One longstanding theory suggests that itching can be elicited by mild pain, or more specifically, that an itching sensation occurs when weak signals are transmitted from group C fibers. We consider that this theory can be rejected for itching related to herpes zoster. Recently, the existence of a specific neural pathway from the skin to the thalamus has been suggested to explain itching, and it is considered possible that pain and itch may have different pathologies. This view has been substantiated in successive recent studies. Mice with TRPV1 exclusively expressed in MrgprA3^+^ neurons exhibited behaviors indicating only itching and no pain in response to capsaicin [10]. Another recent study demonstrated that neuropeptides are related to itching. Natriuretic polypeptide b is released from the central terminals of peripheral nerves to stimulate natriuretic peptide receptor A-expressing spinal neurons [11]. This research concerned the cellular/molecular level and did not specifically address the issue of herpes zoster itch; however, we consider that herpes zoster itch may be triggered through a different neural pathway than that of pain.

Zoster-associated pain is considered to result from inflammation of the nerves and accompanying nerve damage and is mainly treated by addressing neuropathic pain. Conversely, PHI lacks an established therapeutic approach. It is frequently managed with neuropathic pain-focused therapies based on the thinking that pain and itch are similar, but some patients have not responded to these treatments. If pain and itch have different pathologies, then a therapeutic approach focusing on itch is required. In this study, we set out to investigate the nature of pain characteristically experienced by PHI patients and surveyed patients with a goal of establishing a therapeutic approach for PHI. As part of the study, we assessed pain using the PDQ, a questionnaire used in screening for neuropathic components; however, we could not demonstrate significantly high PDQ scores in PHI-positive patients. More specifically, our results do not show widespread pain of a neuropathic nature. Itch intensity showed an association with PDQ scores only in the acute stage, when a moderate, positive correlation was observed. However, no correlation was noted in the other stages. To elaborate, PHI patients do not necessarily have a neuropathic component to their condition, and neuropathic pain therapies may not be effective against PHI in some cases.

We did not investigate synchronism for pain and itch in this study. Thus, we believe further investigation is needed. The occurrence of itch in pain-free intervals would seem to suggest that the neural pathways for pain and itch probably differ. At the very least, we believe that the nature of neuropathic pain is not a characteristic component of PHI and that pain and itch accordingly require different therapeutic approaches.

## 5. Conclusion

We investigated the relationship between pain and itch in herpes zoster. We could not demonstrate a large neuropathic component for PHI in herpes zoster patients; pain and itch may have different mechanisms. Thus, PHI-focused therapies are needed, and we anticipate further research to establish such therapies for PHI.

## Figures and Tables

**Table 1:** Background.

	*n*=76	Acute stage		Subacute stage		Chronic stage	
		PHI (+), *n*=47	PHI (−), *n*=29	PHI (+), *n*=28	PHI (−), *n*=48	PHI (+), *n*=34	PHI (−), *n*=42
Age	65.4 ± 14.7	63.3 ± 15.5	68.9 ± 12.6	65.0 ± 15.7	65.7 ± 14.2	62.7 ± 14.7	67.6 ± 14.4
Sex (male : female)	27 : 49	17 : 30	10 : 19	10 : 18	17 : 31	10 : 24	17 : 25
** **Trigeminal nerve	13 (17%)	6	7	7	6	8	5
** **Cervical nerve	17 (22%)	14	7	9	12	10	11
Site							
** **Thoracic nerve	31 (41%)	16	11	9	18	9	18
** **Lumbosacral nerve	15 (20%)	11	4	3	12	7	8

Age: mean ± standard deviation; sex and site: no. of patients.

**Table 2:** Therapeutic regimens.

	*n*=76 (multiple choice)	Acute stage	Subacute stage	Chronic stage
		No of patients (%)	No of patients (%)	No of patients (%)
1	Tricyclic antidepressants	1 (1%)	16 (21%)	6 (8%)
2	Calcium channel *α*_2_-*δ* ligands	29 (38%)	53 (70%)	31 (41%)
3	Extract of inflamed cutaneous tissue from rabbits inoculated with vaccinia virus	4 (5%)	8 (11%)	1 (1%)
4	Duloxetine	0 (0%)	4 (5%)	2 (3%)
5	Mexiletine	0 (0%)	0 (0%)	1 (1%)
6	Narcotic analgesic	3 (4%)	16 (21%)	11 (14%)
7	Tramadol hydrochloride/acetaminophen	4 (5%)	4 (5%)	3 (4%)
8	NSAIDs/acetaminophen	20 (26%)	1 (1%)	1 (1%)
9	Others	2 (3%)	2 (3%)	4 (5%)
10	Nerve block	33 (43%)	25 (33%)	2 (3%)

NSAIDs: nonsteroidal anti-inflammatory drugs.

**Table 3:** Prevalence of PHI.

	*n*=76	Acute stage	Subacute stage	Chronic stage
		No of patients (%)	No of patients (%)	No of patients (%)
PHI	Total	47 (62%)	28 (37%)	34 (45%)
Severity of itch	Mild^*∗*^	23 (49%)	24 (86%)	27 (79%)
	Moderate^*∗∗*^	13 (28%)	3 (10%)	5 (15%)
	Severe^*∗∗∗*^	11 (23%)	1 (4%)	2 (6%)

PHI (+): strength scale ≥1; itch severity: 11 grades of severity on a scale from 0 to 10; 0 = none; 10 = worst itching possible; ^*∗*^mild (rated as 1–3 on a scale of 0–10); ^*∗∗*^moderate (rated as 4–7 on a scale of 0–10); ^*∗∗∗*^severe (rated as 8–10 on a scale of 0–10).

**Table 4:** Time of itch onset.

	*n*=76	No of patients (%)
1	Only during the acute stage	17 (22%)
2	Only during the subacute stage	2 (3%)
3	Only during the chronic stage	6 (8%)
4	All stages	12 (16%)
5	Acute + subacute stages	9 (12%)
6	Subacute + chronic stages	5 (7%)
7	Acute + chronic stages	9 (12%)
8	No itching at any stage	16 (21%)

**Table 5:** Correlation between PHI and PHN in the chronic stage.

*n*=76	PHI (+)	PHI (−)
	No of patients (%)	No of patients
PHN (+)	11	14
PHN (-)	23	29\8

PHI, postherpetic itch; PHI (+), strength scale ≥1 in the chronic stage; PHN, postherpetic neuralgia; PHN (+), VAS ≥30 mm in the chronic stage.

**Table 6:** Comparison of scores at each stage by PHI.

*n*=76	Acute stage			Subacute stage			Chronic stage		
	PHI (+), *n*=47	PHI (−), *n*=29	*p*	PHI (+), *n*=28	PHI (−), *n*=48	*p*	PHI (+), *n*=34	PHI (−), *n*=42	*p*
VAS	71 (41)	72 (4)	0.83	23.5	27.5 (49)	0.59	13.5 (28)	6 (39)	0.23
PDQ	16 (11)	15 (11.5)	0.50	9 (7.5)	9 (11.8)	0.96	8 (6)	4.5 (12)	0.09

Median (interquartile range); *P*: Mann–Whitney *U* test; PHI: postherpetic itch; PHI (+): strength scale ≥1; VAS: visual analog scale; PDQ: painDETECT questionnaire.

## Data Availability

The data used to support the findings of this study are available from the corresponding author upon request.
